# Viscoelasticity Acts as a Marker for Tumor Extracellular Matrix Characteristics

**DOI:** 10.3389/fcell.2021.785138

**Published:** 2021-12-07

**Authors:** Claudia Tanja Mierke

**Affiliations:** Faculty of Physics and Earth Science, Peter Debye Institute of Soft Matter Physics, Biological Physics Division, University of Leipzig, Leipzig, Germany

**Keywords:** matrix mechanics, confinement, extracellular matrix, homogeneities, viscoelasticity, collagen, hydrogels, cancer

## Abstract

Biological materials such as extracellular matrix scaffolds, cancer cells, and tissues are often assumed to respond elastically for simplicity; the viscoelastic response is quite commonly ignored. Extracellular matrix mechanics including the viscoelasticity has turned out to be a key feature of cellular behavior and the entire shape and function of healthy and diseased tissues, such as cancer. The interference of cells with their local microenvironment and the interaction among different cell types relies both on the mechanical phenotype of each involved element. However, there is still not yet clearly understood how viscoelasticity alters the functional phenotype of the tumor extracellular matrix environment. Especially the biophysical technologies are still under ongoing improvement and further development. In addition, the effect of matrix mechanics in the progression of cancer is the subject of discussion. Hence, the topic of this review is especially attractive to collect the existing endeavors to characterize the viscoelastic features of tumor extracellular matrices and to briefly highlight the present frontiers in cancer progression and escape of cancers from therapy. Finally, this review article illustrates the importance of the tumor extracellular matrix mechano-phenotype, including the phenomenon viscoelasticity in identifying, characterizing, and treating specific cancer types.

## Introduction

### The General Phenomenon of Viscoelasticity

In the nature, certain materials undergo deformations other than purely elastic ones, where the material will fully return to its original shape upon the removal of the external force. Among them are viscoelastic materials. Viscoelastic materials, according to their name, unite two distinct characteristics. The “viscous” part means that they deform gradually when subjected to an applied external force. The “elastic” part means that the material comes back to its original shape after a deforming force is eliminated. In contrary, in pure viscous fluids there is a deformation closely succeeded by a permanent reorganization of the molecules in the fluid. The mechanical characteristics of materials are generally measured in the form of their stress-strain (or load-deformation) response. Specifically, in purely elastic materials, the curves of stress and strain under load and unload are placed on top of each other. In general, the viscoelasticity represents a time-dependent inelastic characteristic of materials, including extracellular matrices frameworks, cells, cell clusters and entire tissues. In specific, the reaction of the material to a stimulus is lagged and a “hysteresis” loop is created, as well as energy is lost within the material. It usually dissipates as heat.

**FIGURE 1:**
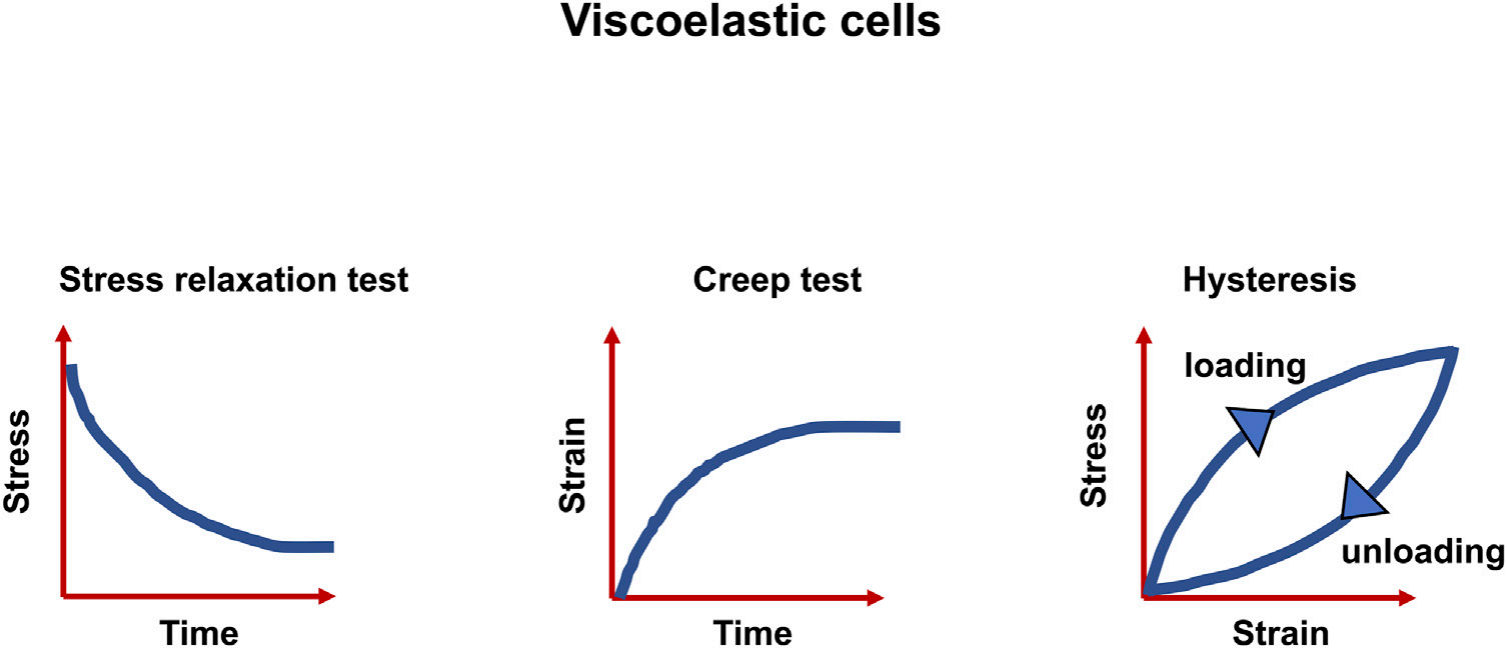
Viscoelastic matter are characterized by three features, the relaxation of stress, the response to creep test and the hysteresis between stress and strain.

A multitude of inelastic characteristics of actual materials are known, among them are viscoelasticity, plasticity and fracture. However, the focus of this review is placed on viscoelasticity. The inelastic response can be witnessed as a slow or partial recovery of the material upon removal of the forces that induced the deformation. Moreover, it is reasonable that the deformation is a function of the history of imposed forces. In summary, viscoelastic materials exhibit three key characteristics: stress relaxation, which means the stress decrease with time (a response of a viscoelastic material to a constant strain step), creep (a constant stress with decreasing strain as a function of time), and hysteresis (a mismatch between loading and unloading processes) (Banks et al., 2011) (Figure 1). As the term “viscoelasticity” implies, this type of mechanical reaction brings together the reaction of elastic solids and viscous fluids. Therefore, it is not only solids but also liquids that are capable of displaying such a characteristic feature. However, the nature of how these materials answer varies greatly. Specifically, the reaction of a fluid to a specific deformation from any two conditions would be identical, whereas a solid, for instance, in its initial shape and after a deformation would react in a different way. Expressed in more general terms, for solids, pure strains can influence the response of the material, however, rotations can have no impact (Truesdell et al., 2004).

Another characteristic of viscoelastic materials is that their mechanical behavior relies on the rate of deformation. The material stiffness rises according to the loading rate. Consequently, there is no single stress-strain curve, but a range of curves depicting the deformation response at various deformation rates. In more detail, viscoelastic properties usually arise on a variety of time scales (relaxation times) in the selfsame material. The stimulus responding at short or very short relaxation times (shorter than 1 s) needs to be examined in dynamic conditions using an oscillating excitation at a constant frequency or over a band of frequencies. The behavior at high relaxation times (from 1 s to hours) can be probed through creep experiments (a load is imposed and held constant throughout while the deformation is monitored) or relaxation experiments (the material is stretched and held at constant strain during which the stress is monitored in time). In this review article, an emphasis is placed on the viscoelastic behavior of the tumor extracellular matrix, which reacts as solids.

## Concepts of Linear and Nonlinear Viscoelasticity

Although the traditional linear theories of solid mechanics can be extended to a larger class of materials, because many different nonlinear constitutive equations can in fact have the identical linear first approximation (Truesdell et al., 2004), the majority of natural phenomena are nonlinear. Consequently, nonlinear theories are capable of yielding far more precise answers to the actions of materials including living matter, such as extracellular matrices, cells and tissues.

### Concept of Linear Viscoelasticity

A displacement is defined as elastic when the undeformed shape is fully restored after elimination of all external forces (Terzopoulos and Fleischer, 1988). The fundamental assumption underpinning the constitutive laws of conventional elasticity theory is that the stress-strain relationship is the same for both loading and unloading, and that the restoring force (stress) is a univalent function of the actual deformation (strain), not its antecedent. To quantity elastic restoring forces, it is feasible to utilize displacement potential energies, what is the characterization that is used in the formulation of those models. Similar to an ideal spring, an elastic model material accumulates potential energy while deforming itself and fully releases the energy when it resumes the former shape. In contrast, a perfect (Newtonian) fluid holds no deformation energy; therefore, it displays no elasticity. Hence, the interest is on models depicting this very common inelastic deformation phenomenon, which lies between fully elastic solids at one side and viscous fluids at the other side. Specifically, the case where the relationship between the stress, strain, and strain rate is nonlinear is a matter of interest in future approaches and thus represents an important Frontier in the field of cancer research. For extracellular matrix scaffolds, cells, clusters of cells and entire tissues, the pure linear elastic behavior cannot be detected. However, the majority of the material can be inferred to be linearly viscoelastic in response to small deformations.

### Classical Concept of Nonlinear Viscoelasticity

**FIGURE 2:**
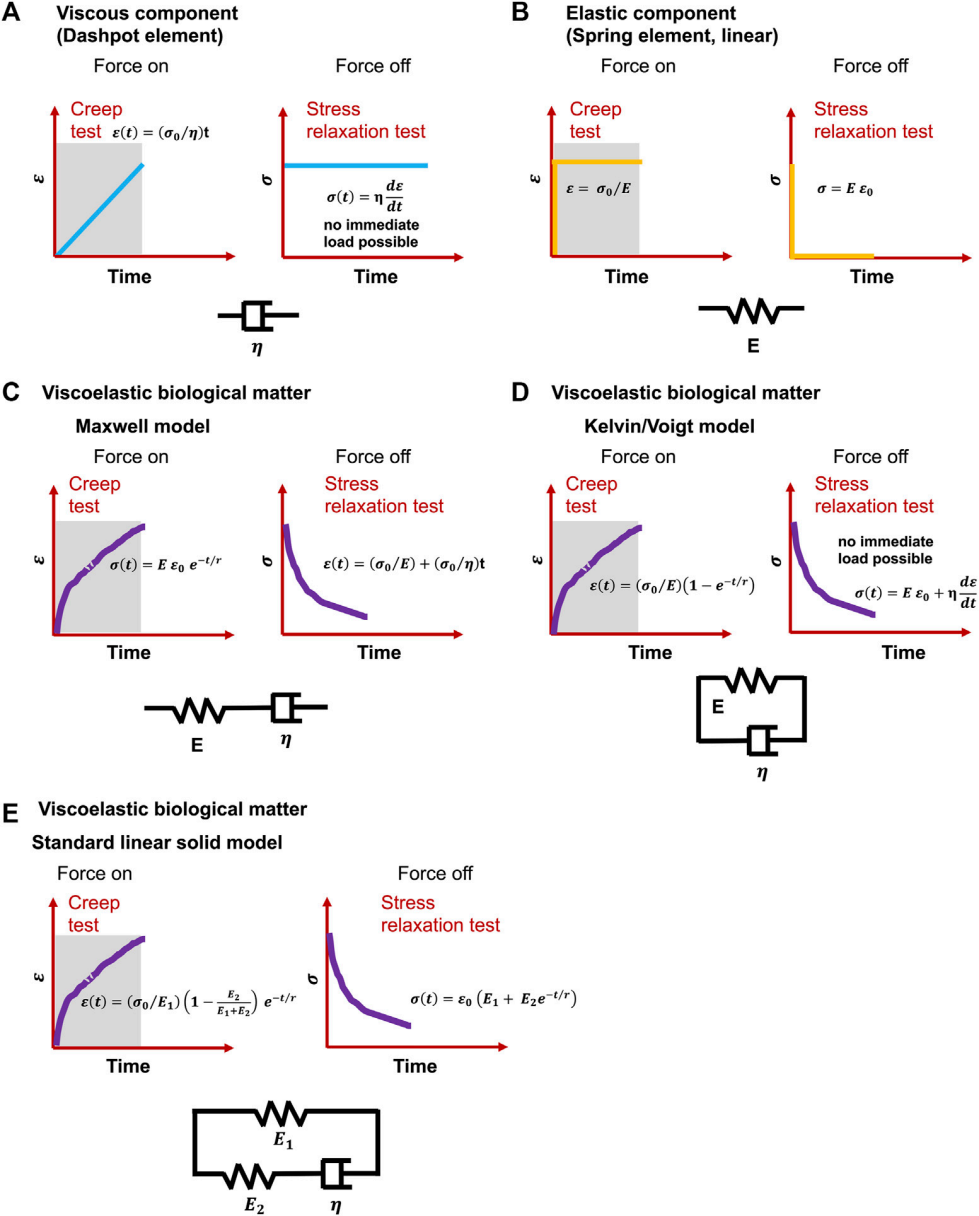
Classical viscoelastic models for creep and stress relaxation testing caused by immediate and constant stress $\sigma_{0}$ and strain $\varepsilon_{0}$ . **(A)** Spring element, **(B)** dashpot element, C-E) Cells undergo a material response when they are mechanically reshaped (deformed): **(C)** Maxwell model, **(D)** Kelvin or Voigt model and **(E)** Standard linear solid model. *t* is time, E represents the elastic modulus, η is the viscosity of dashpots and r denotes relaxation time.

Even though definitions differ, viscoelasticity is a common characteristic of materials that, when deformed, exhibit both viscous (dashpot-like) and elastic (spring-like) features (Figures 2A,B) (Şengül, 2021). Corresponding to the influence of time on their mechanical response, viscoelastic materials can also be designated as time-dependent materials (Banks et al., 2011). The experimental analysis of this kind of materials is basically more complicated in relation to time-independent materials, since it is not feasible to hold time constant or to remove it in the course of an experiment (Findley et al., 1976). Moreover, the nonlinear viscoelastic behavior of cells has been attributed to cytoskeletal tension (Kollmannsberger et al., 2011). Although observational evidence for cell viscoelasticity has been noted since earlier biomechanical efforts (Y. Fung, 1967; Y.-C. Fung, 1993; Levin and Wyman, 1927), it has been disregarded in benefit of solely hyperelastic approaches because of the emphasis on quasi-static analyses, limited existing experimental data, and mathematical considerations. In fact, whereas hyperelastic modulations have dominated the investigation of biomaterials, viscoelastic effects including stress relaxation, creep, hysteresis, and variable frequency behavior are frequently overlooked (Zhang et al., 2021). Viscoelastic modeling has been first explored by merging linear rheological elements that possess strictly elastic or strictly viscous behavior-springs and dashpots (Levin and Wyman, 1927). In the most basic and oldest versions, these two elements are connected in series, termed Maxwell model (Figure 2C), or in parallel, termed Kelvin-Voigt model (Figure 2D) (Christensen, 1980). To account for physiological effects, these models are augmented by a broader range of rheological elements, such as the standard linear model (Figure 2E) (synonymously referred to the Zener model) or the generalized Maxwell model (Brazel et al., 2012). The application of these linear models to nonlinear viscoelasticity has also been conducted by applying different generalizations (Monsia, 2011; Balbi et al., 2018). These viscoelastic based formulations afford a basis for precisely determining the velocity-dependent characteristics of living tissues.

### New Concept of Nonlinear Viscoelasticity Model

**FIGURE 3:**
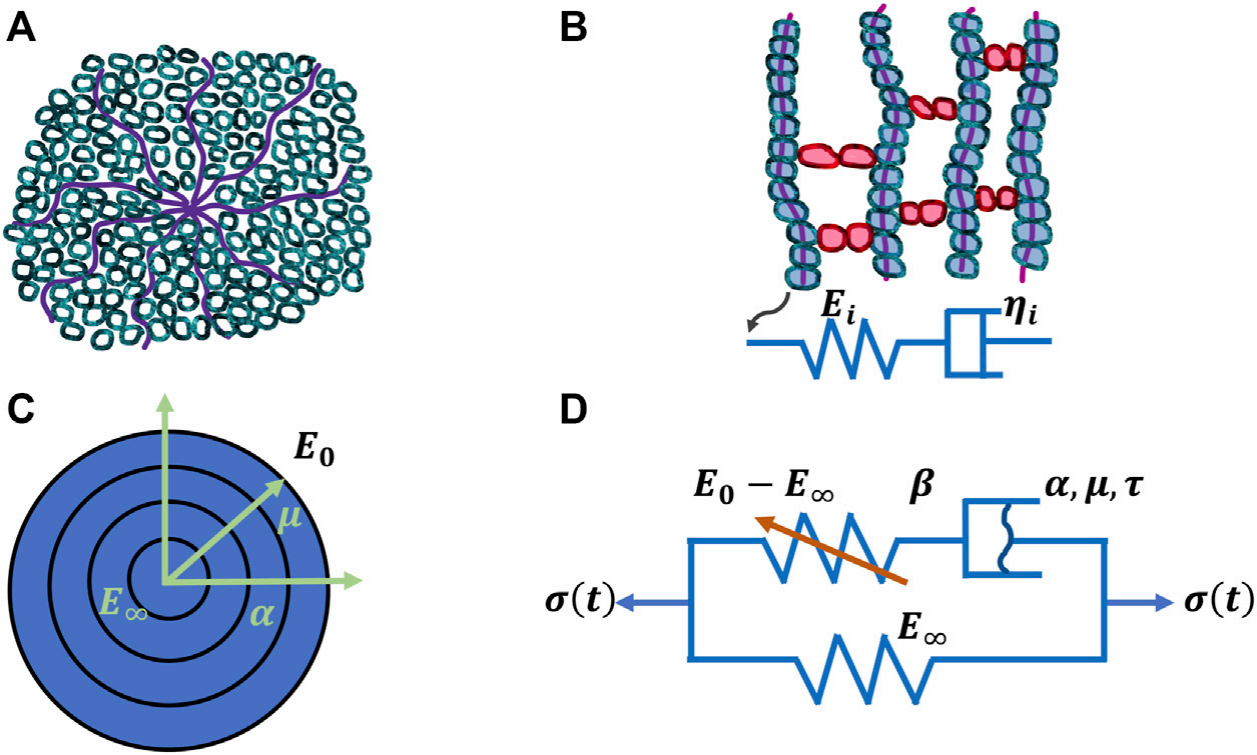
Four models for viscoelasticity. **(A)** Classical arm-retraction model, **(B)** Classical Prony series model, **(C)** New model and **(D)** Nonlinear spring-dashpot model.

The mechanical properties of materials, such as stiffness, alter with temperature. Therefore, it stands to reason that the deformation of viscoelastic materials also changes with temperature. As a matter of fact, the deformation of viscoelastic matter relies on the temperature with the occurrence of a thermal transition. Alterations of the free volume or relaxation time are employed to explore this transition characteristics (Q. Xu et al., 2020). The viscoelastic deformation has been explained in terms of two atomistic phenomena (Roylance et al., 2001): on the one hand, the distortion of the lengths and angles of chemical bonds linking atoms in a small and fast agitation, and on the other hand, the large-scale redistributions of atoms and molecules. Thermal transitions at increased temperatures can encompass a handful of steps, such as for polymers, γ, which involves the local movement of molecular bonds, and β, which denotes the bending and stretching events of molecular bonds, glass, which encompasses the transition from the glassy to the rubbery phase, and terminal transitions that occur from melts into liquids (McCrum et al., 1991). Various transitions exist for diverse materials, but the glass transition is the principal type of deformation for viscoelasticity, which becomes the subject of attention in several respects. The viscoelasticity of materials can be denoted through the relaxation modulus E(t) or the dynamic modulus E*(ω) = E′(ω) + iE′′(ω). E(t) and E*(ω) rely both on temperature which can be transferred into a time or frequency equivalency based on the time-temperature superposition concept. Several biophysical models have been proposed to grasp the linear viscoelastic characteristics. For example, the Rouse model (Rouse, 1953) is built on molecular dynamics theory and employs Brownian motion theory to predict single-chain diffusion of beads joined together through harmonic springs. Moreover, the Kremer-Grest model (Kremer and Grest, 1990) utilizes up to several hundreds of chains (Likhtman et al., 2007) for probing the individual polymer entities. The single-chain theories including the tube theory (de Gennes, 1971) and the arm retraction model incorporating arm launch (de Gennes, 1979) (Figure 2) have also been employed to characterize the linear viscoelastic performance of entangled polymers, such as hydrogels composed of extracellular matrix molecules. The generalized Maxwell model seems to be the most frequently used model for characterizing the glass transfer of linear viscoelastic solids that can also be applied to tumor extracellular matrix scaffolds. For the unwrapped polymers, the spring-pot row of the generalized Maxwell model physically depicts various molecular chains possessing different lengths with time distributions (Roylance et al., 2001) (Figure 3). E(t) of generalized Maxwell model can be denoted as provided in the following Equation 1:

$$E\left(t\right)=\;E_{\infty }+\sum_{i=1}^{n}E_{i}e^{-\frac{E_{i}}{\eta_{i}}t}$$ (1)

where $E_{\infty }$ represents the modulus at infinite time, $E_{i}$ stands for the elastic modulus of the spring, $\eta_{i}$ is referred to as viscosity of the linear dashpot in series, and *n* denotes the number of spring-dashpot terms. This merged exponential term in a single discrete spectrum is also known as the Prony series. It merges toward a standard solid model when n is equal to 1. The Maxwell or generalized Maxwell-based model along with its Prony series formula are widely adopted to match the modulus of linear viscoelastic materials (Soussou et al., 1970). These materials encompass polymers (Krairi and Doghri, 2014), dielectric elastomers (O’Brien et al., 2009; Bai et al., 2014), glasses (Koontz et al., 2012), silicon (Norris, 2012), tissue (Eastwood et al., 2015), blood vessels (Singh et al., 2003), brain (Elkin et al., 2011), ligament (Provenzano et al., 2002), worm (Backholm et al., 2013), and asphalt concrete (AC) (Q. Xu and Solaimanian, 2009). The generalized Maxwell model has also been employed as a foundation that can be broadened to plasticity models which comprise the viscoplastic Bingham-Maxwell model (Prior et al., 2016). Additional models similar to the generalized Maxwell model encompass the so-called generalized Kelvin model and the Burgers model (Chaudhuri et al., 2015). The Prony series is mathematically elegant with its exponential time-integration equation (Slanik et al., 2000), and has therefore been adopted as the standard material model in mainstream numerical programs, such as ANSYS and ABAQUS, for the purpose of modeling material and structural behaviors. The nonlinear viscoelastic models have also been advocated for the modeling of materials with high deformations or characteristics that evolve with deformation or time (Kim, 2011; Xu and Engquist, 2018). One of them is the model of Schapery that regarded the elastic modulus of the spring as a nonlinear function of time (Schapery, 1969), and several other models have estimated the stress of the dashpot in a nonlinear function to be dependent on either strain rate (Monsia, 2011; Chung and Buist, 2012) or relaxation time (Xu and Engquist, 2018). However, liquid models are usually not included.

Nevertheless, there are some critical concerns that have emerged. The models based on the theory of multiple molecular chains have been deduced and confirmed principally for polymers, and they are less appropriate to characterize the physics of other compounds with a dissimilar morphology for instance, materials with an amorphous structure. For the generalized Maxwell model or Prony series: one issue is that the Prony series formula can generate instability in fitting experimental acquisition data. A second issue is that it is quite hard to specify a huge number of model inputs based on experimental observations. A third issue is that with a large number of model inputs, the precision for fitting experimental data has increased in mathematical terms. But the physical explanation of this large spring and dashpot arrangement turns out to be less obvious and more complicated (Q. Xu et al., 2020). These inadequacies of available models are the motivation to strive for the creation of a new viscoelastic material model that more precisely depicts a broad spectrum of materials (Q. Xu et al., 2020). In specific detail, a theoretical model and a mathematical solve have been formulated. Experimental confirmations have been carried out for a variety of materials that span from inorganic materials to biomaterials.

In briefly, the model has been found to increase precision both in fitting experimental data and in forecasting out-of-experimental-range moduli. The model is both numerically stable and will not slow down the computational process. It employs fewer model variables than the generalized Maxwell model or the Prony series. In the proposed model, the nonlinear strain-hardening characteristics are also taken into account. Hence, the novel Xu’s model (Q. Xu et al., 2020) is debated in the following. An innovative nonlinear viscoelastic model for describing the glass transition of solid matter has been introduced to surmount the inadequacies of available models. The model characterizes the modulus with merely five to six variables, but without an additional variable to account for nonlinear hardening, model variables in a continuous range. Experimental validations on several kinds of materials have revealed that the new model has improved fidelity both in fitting the experimental measurements and in forecasting the relaxation modulus beyond the experimental regime, compared to the generalized Maxwell model or the Prony series, which is the most commonly employed model for solid matters. Accurate forecasting of the modulus might be extremely beneficial, since laboratory assays can only cover a narrow band of decreased frequency or time. Moreover, the new model of Xu can predict E(t) beyond the experimental region and is more precise with a smoother curve compared to the Prony series. The forecasted curve records the glass transition more obviously and uniformly than the Prony series. In contrast, the Prony series attempts to provide an exact fit just to existing experimental data, but it can either overestimate or underestimate modulus levels beyond the experimental region, probably leading to erroneous E_0_ or E_∞_ values. The competition between the new model and the Prony series (or the generalized Maxwell model) is equitable, since the same optimization scheme has been adopted to define/adjust the modelling variables. Using the identical number of model variables, the suggested model obtained a more definite solution compared to the Prony series. With a fairly very high number of the terms of the generalized Maxwell model obtained equal or more than 30, its accuracy for data fitting can be enhanced, whereas it remains elusive whether the data prediction beyond the experimental limit is accurate or not. However, a pre-smooth method can be employed to elevate the fitting precision of the Prony series (Park and Kim, 2001). The new model might be considered superior to the generalized Maxwell model or the Prony series if the following issues are taken into account. Firstly, a model, such as the new one, with fewer model variables that meets the fitting precision is frequently favored for simplicity. Secondly, a high number of model inputs in the Prony series (generalized Maxwell model) can generate more uncertainty with non-unique solutions for the determination of the model inputs utilizing mathematical optimization techniques. Thirdly, it is harder to account for the physical phenomenon for the model with a comparatively much higher number of model variables, apart from improving the precision for fitting the experimental results. Fourthly, the presented new model provides higher flexibility, such as it acts a standard solid-model when α = 1, and fifthly the nonlinear strain-hardening phenomenon can be included in it.

Consequently, the new model can be employed as an alternative technique to account for the viscoelastic properties of a huge spectrum of solid materials to further elevate the accuracy of the prediction of data. The generalized Maxwell model cannot model the specific or abnormal physical characteristics, such as a stress overshot of distinct materials covering structural glasses (Wisitsorasak and Wolynes, 2017) and amorphous solids (Jiang et al., 2015).

## Application of the Viscoelasticity on Cancer

Living matter, including (cancer) cells, tumor extracellular matrices, and entire tissues, represent soft and complex biomaterials. It is therefore a great challenge to characterize them mechanically. However, it has turned out that viscoelasticity is an inherent characteristic of tissues, as with all polymers and elastomers, and can also be impacted through fluid flow across the porous matrix architecture of tissues. Due to this fact, hemodynamics and mechanical properties have to be investigated at the same time, when examining whole tissues. In this regard, even on larger length scales, such as the organic level, viscoelasticity can be detected, since the components of organs exhibit viscoelastic responses. Apart from physiological cell migration, tissue morphogenesis and organ development, the viscoelasticity of extracellular matrices plays a fundamental role in the progression of cancer (Rozario et al., 2009; Tetley et al., 2019). Therefore, the viscoelasticity of tumor extracellular matrices can be modeled firstly, by cell-based and energy minimization in Vertex models (Manning et al., 2010; Staple et al., 2010; Bi et al., 2014) or cellular Potts models (Vroomans et al., 2015) and secondly, by topological models based on cell contact networks of non-confluent tissues, including embryonic and cancerous tissues (Douezan et al., 2011; Mongera et al., 2018, 2021; Petridou et al., 2019, 2021).

However, viscoelasticity in biological environments, including heathy and diseased states, has only recently begun to be considered important. Specifically, the cell mechanics have been linked to multiple human diseases or disease stages (Huang and Ingber, 2005; Lim, 2006; Lee and Lim, 2007; Fuhrmann et al., 2011; Maciaszek et al., 2011; Prabhune et al., 2012; Efremov et al., 2014). For instance, the red blood cells that are infected with malaria raise their stiffness and increase their stickiness, both of which is rather not supportive for the transport of oxygen, and may cause subsequently severe anemia, coma or finally organismic death (Lim, 2006). A similar phenomenon can be seen in solid cancers, where the oxygen transport is often severely impacted by an altered mechanical microenvironment (Le Maout et al., 2020). For this reason, the accurate measurement of the mechanical features of the tumor extracellular matrix (see below) can be very decisive for the diagnosis of human diseases and the improved comprehension of biological processes in cancer, such as metabolism.

In cancer, it is particularly difficult to determine the beginning of a malignant course and to predict it at all. Therefore, the different stages of malignant progression must be characterized by structural, molecular, or mechanical markers. However, there are not many such markers which are then also generally valid. Therefore, the different stages of malignant progression of cancer have been described by general suggestions. Specifically, in cancer disease, the initiation, growth and progression of solid cancers relies on specific hallmarks that had been identified over 2 decades ago in 2000 (Hanahan and Weinberg, 2000) and even refined a decade later then in 2011 (Hanahan and Weinberg, 2011). These milestones still disregard the mechanical properties of the cancer (Mierke, 2014). Specifically, the mechanical properties of cancer cells and their microenvironment should be included as milestones. Solid cancers and cancer cells cannot be treated as isolated entities that only feel their cellular neighbors without any contact to extracellular matrix molecules, embedded factors or embedded other cell type or structural and mechanical cues. Cancer is not just a collection of specific cells that divide, invade, and spread in a random manner. Instead, cancer is a multi-layered accurately fine-tuned event that demands the entire organism, which acts on the process of cancer development and progression. The malignant course of tumor diseases should also be included in the analysis of mechanical characterization. The transformation of a cancer cell from a benign phenotype to an invasive or metastatic entity entails both biological factors, such as up- or down-regulation or inhibition of the expression of certain genes and cancer markers (Vogelstein and Kinzler, 2004; Simpson et al., 2005), and physical elements, such as modifications of cell and tissue architecture (A. Hall, 2009; The Physical Sciences - Oncology Centers Network, 2013; Wirtz et al., 2011). Lately, mechanical alterations of cancer cell phenotype have been conceived as an important part, with changes in cell forces playing a crucial role (Kumar and Weaver, 2009). Hence, it can be deduced that specific hallmarks, termed systemic hallmarks, addressing these points needs to be postulated (Paul, 2020). The organismic level is excluded here and the focus is set towards the structural and mechanical interplay between the tumor extracellular matrix microenvironment and cancer cells. However, tissue-level characteristics, especially those addressing mechanical characteristics of tumor extracellular matrices, are still underrepresented and need special attention in relation to the success of tumor treatments.

Several biophysical analyses have been performed to identify the mechanical properties of individual cells. In most of the studies, the viscoelastic properties of cancer cells have been compared to healthy counterparts or treated with pharmacological substances that impair cytoskeletal component assembly/disassembly or functions. For example, viscoelastic properties of human cancer cells, such as lung, skin, breast and liver, and normal cells have been investigated using micropipette aspiration technique (Xie et al., 2019). It turned out that the cancer cells are more deformable and their viscoelastic parameters are decreased compared to normal cancer cells (Xie et al., 2019). Atomic force microscopy (AFM) has been employed to benchmark the viscoelastic characteristics of human mammary epithelial cells of varying metastatic capacity in both their adherent and suspended contexts (Nematbakhsh et al., 2017). Notably, cell elasticity has been determined by spatial mapping of the elastic modulus with the force indentation technique, and cell viscosity has been measured based on stress relaxation (Nematbakhsh et al., 2017). The dynamic change in cell mechanical properties, such as the elevation in cell deformability, is directly connected to the development of a transformed phenotype from a non-cancerous, benign cell to a cancerous, malignant cell (Ketene et al., 2012). The reduction in the quantity of actin in the cytoskeleton and its organization is linked in a direct way to the alterations in the biomechanical characteristics of the cells.

In cancer disease, the viscoelastic characteristics of mammalian cells seem to rely on the biological state, such as whether the cells are in a more epithelial or mesenchymal state or on the transition from epithelial-to-mesenchymal in the malignant progression of cancer (Y. Yang et al., 2019). Therefore, certain viscoelastic characteristics can turn out to become a reliable and useful physical biomarker for diseases, such as cancer or others, and age-specific changes (Agyapong-Badu et al., 2021; Efremov et al., 2020; K.; Park et al., 2019). All of which can be impacted by the viscoelastic characteristics of the tumor extracellular matrix scaffold or age-associated changes of the extracellular matrix scaffold. Moreover, the viscoelastic properties of cells have evolved as critical biomarkers of disease condition and progression (Bao and Suresh, 2003). The simplest attempt to specify viscoelastic characteristics of cells explores two main parameters: Stiffness and viscosity, which typify the elastic and dissipative nature of a cell’s reaction to stress (Moeendarbary and Harris, 2014). Elastic response has been implicated as a marker of cancer cells (Cross et al., 2008) or the metastatic potency of cancers (Xu et al., 2012), and has been strongly connected to cell migration in embryogenesis (Barriga et al., 2018). Cell viscosity has been associated with several biological events, such as the porosity and deformability of erythrocytes (Lim et al., 2006), diffusion (Einstein, 1905; Wojcieszyn et al., 1981), and the condition of cells in disease (Eze, 1992; Zakim et al., 1992). In addition, the viscoelastic properties of the tumor extracellular matrix environment may on top alter the viscoelastic response of cancer cells, since these cells are in direct interplay with the tumor microenvironment.

Specific advances have been made in the study of cancer cell migration and invasion: The simple investigation of isolated of cancer cells has been overcome by advancing cellular assays for analyzing cancer cell behavior and function *in vitro* models from simple 2D models without a suitable tumor microenvironment to a more sophisticated 3D microenvironment. The behavior indicates that the tumor extracellular matrix is considered important and therefore, in addition to structural, mechanical characterization seems to play a role. In this regard, the tumor extracellular matrix is changed at the different levels, such as biochemical, architectural, biomechanical and topographical length scales, and therefore, there is an exponential raise in studies that incorporate the matrix in solid tumors (Bissell et al., 1982; Herbison et al., 2019; Cox, 2021).

Another step in the right direction is the analysis of the dynamic performance of cells rather than just endpoints that further advances these 3D assays to 4D assays. For the dynamic analysis of the mechanical properties, the viscoelastic behavior of the tumor environment and that of the cancer cells is of special relevance. Moreover, the mutual interaction between the tumor microenvironment and cancer cells must be considered, which requires simultaneous analysis of structural and mechanical phenotypes (Mierke, 2019, 2020). By characterizing the tumor extracellular matrix environment, an emphasis is on the phenomenon of viscoelasticity and hence an introduction to it is provided in the following section.

From a physical point-of-view, growth, migration and invasion, intravasation, blood or lymphoid circulation, arrest/adhesion, and extravasation of cancer cells demand distinct cell-mechanical characteristics that contribute to the survival of cancer cells and subsequently full execution of the metastatic cascade. In this regard, metastatic cancer cells are generally softer than their non-malignant equivalents (Guck et al., 2005; Fischer et al., 2017; Lv et al., 2021), and high deformability of both the cell and the nucleus is hypothesized to confer a substantial benefit in terms of the metastatic status (Fischer et al., 2020). Nevertheless, it remains ambiguous whether there is a more fine-tuned but steady mechanical state that accounts for all the mechanical characteristics necessary for survival across the cascade, or whether cancer cells must dynamically fine-tune their characteristics and intracellular constituents at every new stage.

## Viscoelastic Properties of the Tumor Extracellular Matrix

The viscoelasticity of the tumor extracellular matrix arises due to covalent nature of crosslinking that considers the extracellular matrix as an elastic-like network (Muiznieks and Keeley, 2013) and the strain-stiffening response of collagen scaffolds, which emerges from the network level and specifically its connectivity (Wen and Janmey, 2013; Han et al., 2018; Jansen et al., 2018). The nonlinear characteristics of tumor extracellular matrices emerges from strain of only 10% increase where stiffness increased by 100 times before a rupture of the scaffold occurs (Sharma et al., 2016).

However, the tumor microenvironment is not simply a pure matrix scaffold. Instead, the tumor microenvironment represents a dynamic tapestry of cancer cells enclosed by the extracellular matrix and a plethora of stromal cells, among them fibroblasts, hematopoietic and lymphoid cells, immune cells, and multiple tissue-specific cells, including adipocytes, endothelial cells and pericytes. Due to the malignant transformation of the normal tissues to cancerous tissues, such as the progression of the primary solid tumor to cancer cell invasion, cancer dissemination, and consequently metastasis, the mechanical characteristics of the tumor are largely impacted (Kumar and Weaver, 2009; Gensbittel et al., 2021). These tumor microenvironment alterations are driven by increased contractility of cancer cells, the enlargement of the expanding tumor mass, and changes of the material characteristics of the local tumor extracellular matrix components including viscoelastic properties. Consequently, the physical characteristics of a tissue, such as the stiffness and structure of the extracellular matrix, potentially exert a pervasive impact on cell performance and, in the end, on tissue organization and function. Simple experiments with substrates of different stiffness to which normal thyroid cells and thyroid cancer cells have been attached yielded different results. Normal thyroid cells adjusted their mechanical characteristics to substrates with varying stiffness, while cancer cells were influenced less by the stiffness of the microenvironment (Rianna and Radmacher, 2017). Therefore, it seems important to select not pure elastic substrates but viscoelastic substrates that better represent the natural tumor environment in order to study the cancer cell response to them.

The characterization of viscoelastic materials can be performed in multiple ways that depends on the different exertion of stimuli, such as stepwise testing (creep and stress relaxation), ramp-type testing or sinusoidal testing.

### Stepwise Testing (Creep and Stress Relaxation)

In the past decades, various biophysical approaches have been designed to quantitatively examine the viscoelastic properties of cells (Addae-Mensah and Wikswo, 2008), encompassing mechanical micropipette aspiration (Hochmuth, 2000), optical tweezers (Hoffman et al., 2006), magnetic tweezing cytometry (Fabry et al., 2003; Hoffman et al., 2006), magnetic tweezers (Kollmannsberger et al., 2011; Mierke et al., 2011) and AFM (Efremov et al., 2020; Fischer et al., 2017; B.; Wang et al., 2013). Compared to a number of these approaches, AFM provides the major benefit of being able to directly probe living cells under their physiological constraints with a force and spatial resolution on the scale of piconewtons and nanometers, respectively. The simplest way to describe the mechanical properties of viscoelastic materials is stepwise stimulation, such as creep and stress relaxation experiments. The steps can be performed as single measures or they could be performed as multiple measures with the same force or varying force, such as descending or ascending.

### Sinusoidal Testing

Apart from a single step or multiple step probing, a range of frequencies can be applied. The deformation of the material is recorded in reaction to a sinusoidal load at a specific frequency, where the range of amenable frequencies is delineated by the capacity of the instrument employed, ranging characteristically from 0.1 to 200 Hz for most standard commercial AFMs (Alcaraz et al., 2003; Lu et al., 2006). Typically, measurements of viscoelasticity with AFM can be classified crudely into vibrational (oscillatory) frequency measurements (Chyasnavichyus et al., 2014) and time-dependent measurements of penetration depth, such as stress relaxation (Chyasnavichyus et al., 2014). In particular, the measurement of oscillatory frequencies is the most widely employed, but measurements in liquids are subject to hydrodynamic forces that are heavily impacted by the experimental conditions (Radmacher et al., 1996; Alcaraz et al., 2002).

More recently developed high-speed AFMs possess up to 100 kHz (Nia et al., 2013; Rigato et al., 2017). In distinction, time-dependent mechanical AFM experiments utilize a quasi-steady-state stress-relaxation approach, but this necessitates a fit of the force-impact curves with a predetermined phenomenological model to identify the “pseudo” material properties that delineate the viscoelastic reaction of the cells (Fischer-Cripps, 2004; Darling et al., 2007). In principle, these variables vary depending on the experimental procedure, such as holding time, and are subject to fitting mistakes related to the selected models and the estimation of the unknown variables (B. Wang et al., 2013). Recently, a novel AFM microrheology method has been developed to identify the linear viscoelastic characteristics of complex materials and living cells across five continuous frequency decades, such as 0.005–200 Hz, based on a simple stress relaxation nanoindentation sensing with a standard AFM instrument (Chim et al., 2018). Additionally, the experimental measurements can be immediately analyzed avoiding the requirement to interpret the experimental measurements with any pre-conceived viscoelastic model. These findings are in perfect accordance with traditional oscillatory bulk rheology tests in hydrogels. Apart from AFM, the optical tweezer can be employed to determine oscillatory measurements of intracellular components within cells and a single-cell parallel plates rheometer to probe overall cellular mechanics including viscoelasticity in an oscillatory manner (Mathieu and Manneville, 2019; Alibert et al., 2021).

### Bulk (Tissue) or Local (Cellular) Probing

In addition to the mechanical probing method, the measurements can be performed as bulk or local analysis. An interesting experimental finding is that the mechanical characteristics of the microscale elements of cell and tissue viscoelasticity, such as the components of the cytoskeleton and the cells, do not generally correspond to the macroscale mechanical characteristics of cells and tissues (MacKintosh et al., 1995; Broedersz and MacKintosh, 2014; Petridou et al., 2021). In this way, macroscopic viscoelasticity, such as scaffold geometry and local topology of filaments, often displays nonlinear alterations that are not evident at the microscopic level, such as mechanical characteristics of the biopolymer filaments creating the cytoskeleton. (MacKintosh et al., 1995; Broedersz and MacKintosh, 2014; Mongera et al., 2021). Experimental evidence has been provided for those examples, such as the stiffening response of the cytoskeletal scaffolds (Gardel et al., 2004, 2006; Mierke et al., 2011; Pritchard et al., 2014), phase transitions in the energetic expense of cellular motility (Mongera et al., 2018) or sudden alterations in the viscosity of tissues (Petridou et al., 2019). Therefore, the mechanical loading capacity of individual microscopic elements cannot explain the macroscopic viscoelastic changes. Consequently, the pattern of interaction between the elements must be analyzed. There are at least three theoretical approaches that can be employed for this discrepancy (Table 1).

**TABLE 1:** Three theoretical approaches to describe the discrepancy between macroscopic and microscopic mechanical characteristics of tissues.

Theoretical approach	Model	Description	References
First Approach	Mechanical Model	Microscale basis provides the mechanical characterization of biopolymer filaments	Wen and Janmey (2011); Pritchard et al. (2014)
		Macroscopic viscoelastic features are deduced from the geometry of the framework and local topology of filaments	
		Nonlinear strain-stiffening	
Second Approach	Vertex Model	Microscopic scale serves as the tilting pattern of the elements	Alt et al. (2017); Angelini et al. (2011); Bi et al. (2016); Farhadifar et al. (2007); Merkel and Manning (2018); Park et al. (2015); Pritchard et al. (2014); Sadati et al. (2013) 39–41
		Rheological characteristics, such as stiffness, are obtained based on the energetic expense incurred by the cells as they move through the tissue matrix under their own power	
		Material deformation seem to appear through cell-cell interaction remodeling, such as the nonlinear jamming to unjamming transition	
Third Approach	Network Theory	Analysis of mechanical characteristics across scales	Alvarado et al. (2017); Driscoll et al. (2016); Petridou et al. (2021); Sharma et al. (2016)
		Topology of the scaffold, such as cytoskeleton, fiber networks and cellular networks (spheroids and tissues): how the elements are linked	
		Concept of percolation	
		Percolation is highest at a critical point of stiffness percolation (formation of cracks due to stress action)	

Moreover, the surrounding extracellular matrix cannot simply be considered separately from the embedded cancer cells. In the following, the influence of the viscoelastic tumor environment on the structural and mechanical properties of the cells is discussed.

## Environmental Matrix Viscoelasticity Acts on Cells

Cell and tissue reactions result from forces created by the cell on its own that are opposed by the viscoelastic or active characteristics of the extracellular matrix or ambient cells. Moreover, the extracellular matrix environment can impose mechanical cues on cells and tissues. The focus is placed here on the effect of the environmental matrix on cancer cells. Specifically, the following question is raised: How will cells react when they adhere to surfaces and matrices that withstand deformation, when cells tug or push on them? However, the structural, molecular and mechanical properties of cells seem to be determined by the microenvironment of cells. The response of living cells is well-known to be influenced by both elastic (Discher, 2005; A. J.; Engler et al., 2009) and inelastic (Chaudhuri et al., 2015, 2016) mechanical characteristics of the microenvironment. The inelastic mechanical characteristics of the extracellular matrices can be either viscoelastic or plastic (Wang H. et al., 2014; Liu et al., 2016), where the latter results in long-term non-reversible deformations. Because the architecture and stiffness of the extracellular matrix affect cell spreading, movement, and differentiation (Discher, 2005; A. J.; Engler et al., 2009), the extent of matrix plasticity upon mechanical rearrangement is probably an influential factor in defining cell performance. For instance, the persistent alignment of fibers in the extracellular matrix leads to the persistence of mechanical evidence that can affect the alignment and migration of normal control cells (Dickinson et al., 1994) and cancer cells (W. Han et al., 2016; Provenzano et al., 2008).

Over the course of the last 2 decades, substantial scientific evidence has established that the elasticity or stiffness of the extracellular matrix governs essential cellular processes, among them spreading of cells, cell growth, proliferation, migration, differentiation, and assembly of organoids (Discher, 2005). Linear elastic polyacrylamide hydrogels and polydimethylsiloxane (PDMS) elastomers layered with extracellular matrix proteins are frequently employed to evaluate the effects of stiffness, and it is commonly hypothesized that the outcomes of these types of investigations will mimic the impacts of the mechanical environment encountered within cells *in vivo*. However, tissues and extracellular matrices typically have no linear elasticity (Elosegui-Artola et al., 2014), instead they display much more complicated mechanical characteristics, incorporating viscoelasticity, which is a time-dependent response to strain or deformation, mechanical plasticity and nonlinear elasticity. In the following the intricate mechanical characteristics of tissues and extracellular matrices is presented, the impact of extracellular matrix viscoelasticity on cells is debated, and the possible impact of viscoelastic tumor extracellular matrix scaffolds in cancer treatment is discussed.

Extracellular matrices and entire tissues and cannot be treated as linearly elastic materials because they manifest far more complexity in mechanical response, involving viscoelasticity (Abidine et al., 2021; Martinez-Garcia et al., 2021) mechanical plasticity (Buchmann et al., 2021) and nonlinear elasticity (Elosegui-Artola et al., 2014). Hence, matrix viscoelasticity seems to govern essential cellular processes and can foster types of behaviors not evident in cells that are cultured in purely elastic hydrogels in both two- and three-dimensional culture surroundings (Charbonier et al., 2021). Matrix viscoelasticity seems to be important in revealing the complex interplay between cells and their environment at cell-matrix interaction sites and how these interactions variably impact mechanosensitive molecular signaling paths in cells (Tan and Song, 2021). Specifically, the collagen density can foster the progression of cancer (Provenzano et al., 2008).

**FIGURE 4:**
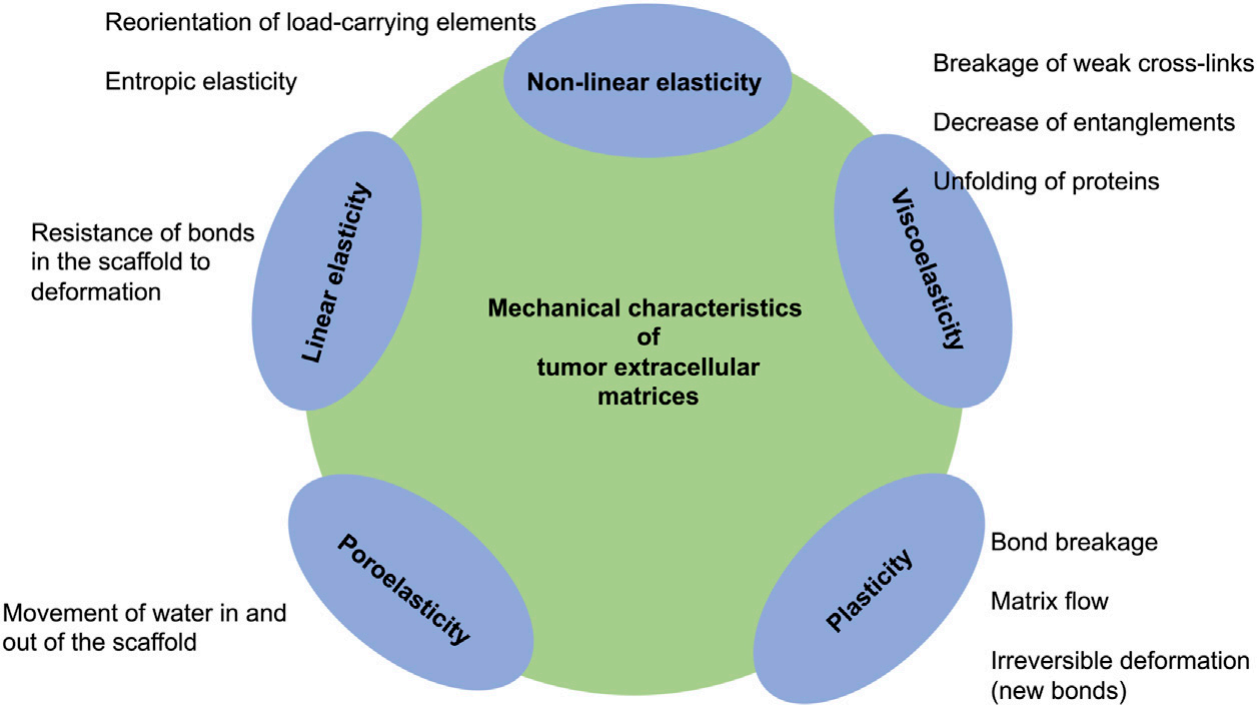
Matrix features of tumor extracellular matrices and biological tissues.

In this context, hydrogels with dynamic characteristics, which are accomplished either through the integration of degradable structural compounds or reversible dynamic cross-links, permit efficient accommodation of cells to the matrix and aid in the achievement of the connected cellular specific functions (B. Yang et al., 2021). Since, it is well known that cancer cells can alter their extracellular matrix environment by secreting of molecules (Dhar et al., 2018; Kano, 2015), release of exosomes (Hoshino et al., 2015), degrading (Stephens et al., 2019), re-orientating (aligning) (B. Lee et al., 2017; Levental et al., 2009; Provenzano et al., 2002; Vader et al., 2009) or cross-linking the matrix scaffold (Levental et al., 2009), it seems to be quite obvious that in turn the tumor microenvironment modifies the properties of cancer cells, including their structural, morphological and mechanical properties. Consequently, many tissues have nonlinear elasticity and do not exhibit the straightforward linear relationship between stress and strain that is typical of most conventional Hookean solid materials, such as concrete or steel. Similar to a nonlinear elastic material, a coiled tether is relatively simple to stretch at first, but gradually tends to get more challenging as it is fully stretched. In particular, networks of cross-linked collagen fibers are assumed to be accountable not merely for tissue viscoelasticity, but also for nonlinear elasticity (Figure 4). In both shear and tensile strains, collagen meshes act similarly to linear elastic materials up to a certain limit of strain; beyond this point, they stiffen as the fibers orient themselves in the direction of peak tensile stress (M. S. Hall et al., 2016; Licup et al., 2015; Steinwachs et al., 2016; Storm et al., 2005; Vader et al., 2009; Wang H. et al., 2014). The alignment of the fibers, such as collagen fibers, can facilitate force transfer over hundreds of micrometers, leading to improved long-range cell communication (Y. L. Han et al., 2018; Wang H. et al., 2014). A theoretical fiber mesh model of collagen has revealed that tight coupling between deformation modes can lead to much higher stiffening of the meshes under triaxial and biaxial tensile loading relative to uniaxial loading (Eckes et al., 2000).

For example, the tumor extracellular matrix environment impacts the cytoskeletons of cells that undergo continuous structural remodeling due to highly dynamic perturbations and fluctuations in their entire life cycle and during the development and progression of cancer. The tumor microenvironment induced cell mechanical characteristics have been attributed to multiple cellular physiological tasks, encompassing cell movement (Lautenschläger et al., 2009; Mierke et al., 2011), differentiation of cells and tissues (Nelson et al., 2006), cell adhesion (Kumar et al., 2006; Qian et al., 2008) and the take-up of nanoparticles through a process termed endocytosis (C. Huang et al., 2013; Wang J. et al., 2014; Wang and Li, 2015). All of which can be severely de-regulated and promote the malignant progression of cancer.

### Effect of the Tumor Microenvironment on the Cell’s Cytoskeleton and Focal Adhesions

Cell proliferation, differentiation and migration rely strongly on the extracellular matrix mechanical stiffness. Natural extracellular matrices are also known to possess dissipative, including plastic and viscoelastic, characteristics that can in turn modify cellular response. There is growing support for the idea that cells can perceive and respond to the physical characteristics of the extracellular matrix, a capability that is key to events such as spreading of cells (Nisenholz et al., 2014; Chaudhuri et al., 2015), cell migration (Trichet et al., 2012; Shenoy et al., 2016; Sunyer et al., 2016), and cell proliferation (Klein et al., 2009; Trappmann et al., 2012; Swift et al., 2013). Focal adhesions, which tether the cell to the extracellular matrix and act as nodes for the replacement of biological and mechanical cues (Parsons et al., 2000; Cao et al., 2015), are generally believed to be responsible for the cellular mechanosensitivity. Consequently, these physiological boundaries between the tumor microenvironment and cancer cells enable the process of mechanotransduction between these two compartments (Boyle and Samuel, 2016). Thereby, the regulation of cells by tumor microenvironments is feasible, such as through signaling via Rho-associated protein kinase (Boyle and Samuel, 2016). Through the continuous interaction between the extracellular matrix scaffold a matrisome is created (Naba et al., 2012). Although the composition, size and remodeling of focal adhesions is altered in 2D and 3D environments (Cukierman et al., 2001), an emerging concept, which is equally amenable to 2D and 3D extracellular matrices, is that stress relaxation of an extracellular matrix can modify the dynamics of adhesion. When a material is subjected to uniform strain, the relative stress reduces with time, potentially a fast or slow acting phenomenon. Focal adhesions are able to react to either rapid or low-speed stress relaxation of hydrogels (Adebowale et al., 2021). Specifically, cells travel at a minimum on substrates with a modulus of elasticity of 2 kPa, which are elastic or display slow stress relaxation, while traveling extensively on 2 kPa substrates, which display rapid stress relaxation (Adebowale et al., 2021). The arrangement and orientation of the cytoskeleton are highly sensitive to the mechanical and structural characteristics of the matrix, such as Young’s modulus, Poisson’s ratio, and roughness (R. De et al., 2007; Wei et al., 2008). Thus, depending on its physical and mechanical properties, a substrate displays various types of characteristics in reaction to cell aggregation (H.-B. Wang et al., 2000). To put it another way: When a substrate is stiff and inelastic, focal adhesions act as structural connections between the extracellular matrix and the actin cytoskeleton (Guo et al., 2006). A focal adhesion provides a stable physical connection that conveys cell adhesion to the substrate (Iwanaga et al., 2001). In contrary, soft and elastic substrates afford a temporary retention in the cellular matrix (Qian and Gao, 2010).

When interfacing with the substrate, cellular reactions, involving relaxation time and adaptation through changes in fibrous structures, are governed through the local deformability of the matrix (De and Safran, 2008; Hsu et al., 2009). The adaptation of the cell cytoskeleton to the mechanical characteristics of the substrate relies on the polymerization and depolymerization of actin fibers (Nekouzadeh et al., 2008), which operate through focal adhesion proteins located at the cell-substrate interface (De and Safran, 2008).

These insights have yielded knowledge of cell-matrix linkages and how these linkages variously modify mechano-sensitive molecular signaling transduction cascades in cells. Beyond that, these findings propose design directions for the next evolution of biomaterials to mimic the tumor extracellular matrix network, with the aim of tuning the mechanics of tissue and extracellular matrix for *in vitro* tissue models for cancer metastasis research.

### Tumor Extracellular Matrix Environment Is Sensed by Cancer Cells

Cells, such as cancer cells, can sense the mechanical stiffness of their microenvironments through by probing the resistance of focal adhesions toward a retrograde flow of actin that is evoked by intracellular contractions based on myosin filaments (Cao et al., 2017; Case and Waterman, 2015; Plotnikov et al., 2012; Shemesh et al., 2005). Focal adhesions, which function as molecular clutches, impact the motion of intracellular structures, such as actin filaments, through offering an adaptable linkages toward the surrounding extracellular matrix (Chan and Odde, 2008; Case and Waterman, 2015). Consequently, the classical motor-clutch model (Chan and Odde, 2008; Bangasser et al., 2013; Bangasser and Odde, 2013) has been proposed that forecasts a biphasic reliance on the cell adhesion-based traction (and subsequently on the spreading of cells) and on the rigidity of the extracellular matrix. Consistent with this, emerging experiments have identified a monotonic enhancement of cell spreading rates along with extracellular matrix stiffness (Ghassemi et al., 2012; Étienne et al., 2015) and this may be linked to reinforcement mechanisms that involve, for instance, activation of adhesion proteins under high environmental stiffness/stress or retention of integrins within the focal adhesions (Elosegui-Artola et al., 2014, 2016).

**FIGURE 5:**
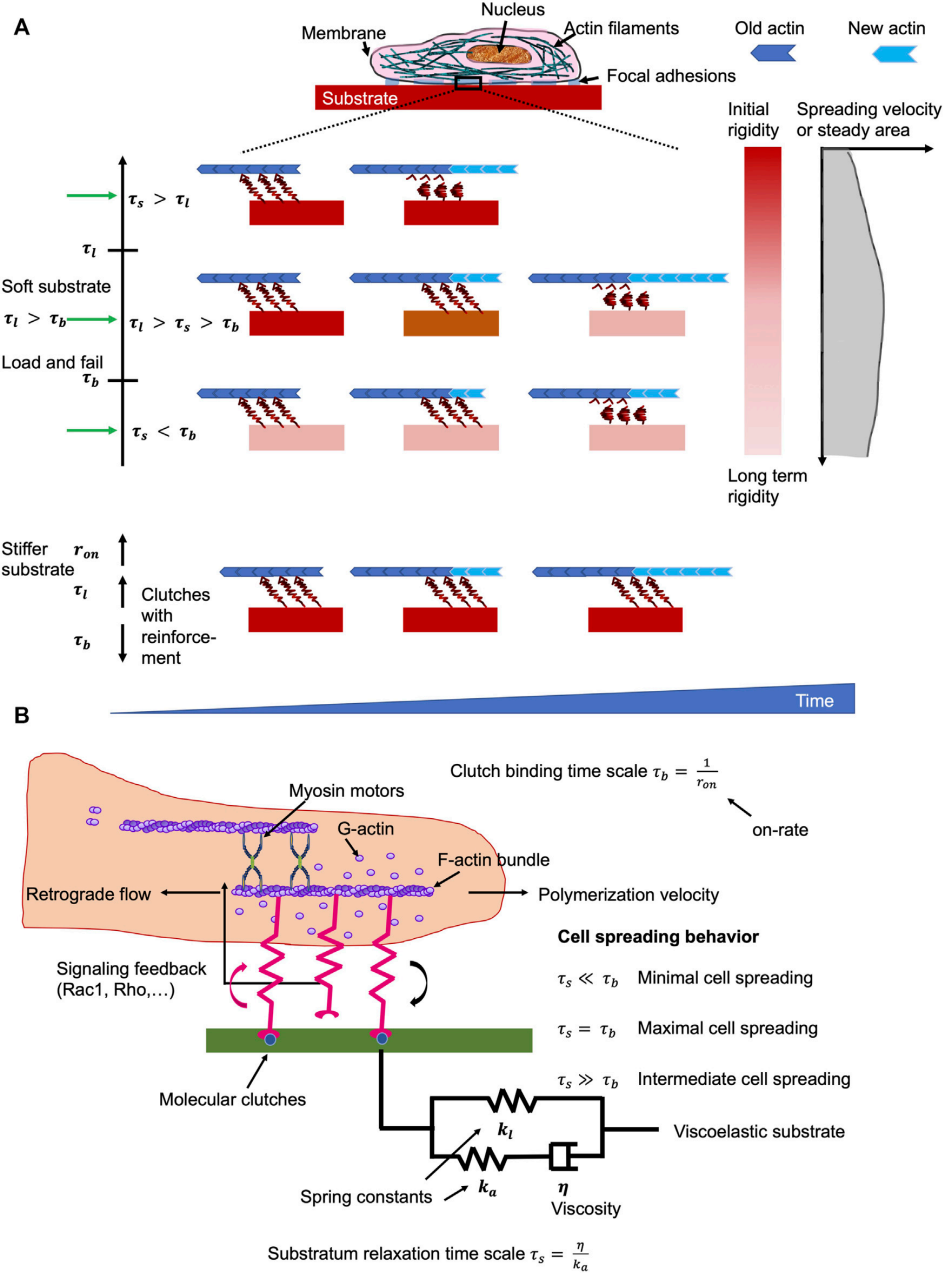
Matrix environments impacts cell mechanics. The ideal viscosity minimizes the retrograde flow of actin whereby the turnover of focal adhesions is prolonged on soft materials. **(A)** Effects if the substrate viscoelasticity toward the characteristics of cells in relation to clutch binding, $\tau_{b}$ , substrate relaxation, $\tau_{s}$ , and cellular life timescales $\tau_{l}.$ In the load and fail domain with $\tau_{l}>\;\tau_{b}$ , the spreading of cells is maximal when $\tau_{l}>\;\tau_{s}\;>\tau_{b}$ . It means that the cells sense a surrounding material with high initial stiffness, which vastly relaxes after the clutch engagement. However, when the viscosity of the material is low, such as $\tau_{s}<\;\tau_{b}$ , cells can merely perceive long-term rigidity that elevates the lifetime of focal adhesions, but did not impair the retrograde flow of actin. When the viscosity of the substrate is relatively elevated, such as $\tau_{s}>\;\tau_{l}$ , the cells perceive solely the starting rigidity, which did not alter the lifetime of focal adhesions, but lead to premature clutches that cannot fulfill their function. In contrast to rigid extracellular matrices, a high number of clutches is created evoked by reinforcement of clutches that cause subsequently prolonged lifetimes of focal adhesions, which restricts the retrograde flow of actin and improves the rate of spreading. **(B)** Sketch of the molecular clutch model that is employed for viscoelastic materials. Optimal cell spreading is gained when the timescale for stress relaxation is close or equal to that of the clutch binding timescale. Out of this balanced state, the cell spreading is impaired.

Besides substrate stiffness, the majority of natural extracellular matrix matters that include biomaterials such as collagen, and fibrin (Roberts et al., 1974), and living tissues (Alcaraz et al., 2003; Deng et al., 2006; Verdier et al., 2009) are inherently viscoelastic and manifest a robust frequency-dependent mechanical responsiveness. Moreover, the spreading of cells can be enhanced through stress relaxation of a cell culture substrate, such as alginate and polyacrylamide, an effect that seemed to rely on the elastic modulus of the substrate (Cameron et al., 2011; Chaudhuri et al., 2015). A local redistribution (causing enhanced ligand density) of the matrix occurred upon deformation to account for this (Chaudhuri et al., 2015), which is consistent with a plastic rather than a viscous reaction. Conversely, experiments have also led to the hypothesis that viscosity has a marginal impact on cell spreading (Chaudhuri et al., 2020). Nonetheless, it is unsure how a merely viscoelastic substrate can have divergent effects on cell spreading, in large part because few theoretical models (Chaudhuri et al., 2015; Gong et al., 2018; De and De, 2019) are in place to clarify the physical mechanisms that direct the cellular reaction to viscoelasticity. To further resolve this crucial concern, a systematic approach to investigate how cell spreading is governed by the viscoelastic constituents of the extracellular matrix has been undertaken by means of analytical mean-field analysis and direct Monte Carlo computational simulations (Van Liedekerke et al., 2015; Chaudhuri et al., 2020). In specifically, by considering the tumor extracellular matrix as a standard linear viscoelastic solid, there is evidence that an intermediate level of viscosity is capable of facilitating cancer cell spreading when the stiffness of the extracellular matrix is fairly weak, which mirrors the circumstance that the substrate relaxation time under such conditions is somewhere between the coupling bond time scale and its typical bond lifetime. In other words, viscosity acts to rigidify soft substrates, which encourages cell adhesion to the extracellular matrix and facilitates consequently cell spreading. As with high stiffness, the large stress carried by the couplings elicits an enhancement of their binding levels as well as an augmentation of integrin tightness (clutch amplification), thereby rendering the cell contribution to substrate stiffness to become a saturated response, and viscosity no longer to be an issue (Figure 5). These datasets can be displayed in heat maps of the propagation response in the parameter volume defined by the substrate and cell time scales.

## Design of Hydrogel Scaffolds That Mimic Tumor Microenvironments

### Mimicking Viscoelastic Characteristics of Tumor Extracellular Matrix Environments With Hydrogels

The extracellular matrix not only offers structural sustenance and governs functional characteristics, but also performs an essential part in tissue physiology through interaction with cells and trafficking of interstitial fluid. The simplest way to mimic the tumor microenvironment appears to be cell culture assays using an extracellular matrix scaffold with the aid of hydrogels. Hydrogels are soft, water-based polymer gels that are increasingly used to fabricate free-standing fluidic devices for tissue and biological engineering applications. Specifically, hydrogel materials and other biopolymer scaffolds can be produced that created almost weak linkages, such as the dynamics of physical cross-links between polymers. For instance, viscoelastic poly ethylene glycol (PEG) hydrogels form dynamic covalent hydrazone bonds, borate bonds or thioester exchange (McKinnon et al., 2014; Brown et al., 2018; Tang et al., 2018). In natural alginate gels, weak ionic cross-linking creates viscoelastic gels (Zhao et al., 2010). Moreover, viscoelastic hyaluronic acid-based hydrogels can be generated through employing hydrazone bonds or guest-host cross-links (Lou et al., 2018; Loebel et al., 2019). Alternatively, weak-bonds can be produced by engineering, namely, the so-called programmed peptide-based hydrogels (Dooling et al., 2016). Weak-bonds within these matrices can be altered in their viscoelasticity independently of their initial elastic modules through the combination of the following elements: molecular weight of the constituent polymer, coupling of inert molecules toward the constituent polymer, which functions as spacers, affinity of weak bonds, relationship between weak and covalent bonds and the total amount of bonds (Chaudhuri et al., 2016; Dooling et al., 2016; Lou et al., 2018; Loebel et al., 2019; Nam et al., 2019; Richardson et al., 2019; Vining et al., 2019). These weak bonds have been reported to be possibly viscoplastic and explore a viscoelastic transition (Müller et al., 1995; Wang and Zocchi, 2011). Single or double networks generated by a combination of covalent and weak cross-links can possibly display viscoplasticity at the bulk scale due to the molecular structure and degradation-evoked alteration of these hydrogels can alter their viscoelasticity (Narasimhan et al., 2021). Specifically, these hydrogels are made of different scaffold structures that offer the accurate guidance and fine-tuning of the dissipation of hydrogen bonding. The hydrogels with adjustable dissipative characteristics are achieved by photopolymerization of a second polymer contained within a preshaped crosslinked hydrogel grid of poly (acrylamide). Specifically, the second networks are prepared with distinct structures and capacities for hydrogen bonding to the first network, which are linear poly (acrylic acid) for the first network and branched poly (tannic acid) for the second network. For example, gels with a second network composed of poly (tannic acid) displayed increased stiffness (0.35 ± 0.035 MPa) and elevated toughness (1.64 ± 0.26 MJ m^−3^) compared to the poly (acrylic acid) counterparts. Moreover, a strategy was outlined for the preparation of hydrogels in which dissipation (loss modulus) can be adjusted separately from elasticity (storage modulus) and which are convenient for cell culture purposes. It can be envisioned that this modular approach to hydrogel fabrication will find uses in customized substrates for cell culture assays and in load-bearing tissue engineering implementations.

Since both the viscoelastic and viscoplastic characteristics of hydrogels can be impacted by poroelastic effects, they have to be taken into account. Tumor extracellular matrix scaffolds display physical interactions of water with other phases that are inevitably to characterize these systems. In specific, the theory of poroelasticity affords a means of delineating the mechanical response based on these interactions, by modeling a porous material that possesses an elastic solid skeleton with fluid-saturated interconnected pores. Using this model, the microstructural variables, phases and interactions, can be compared to scaled-up continuum mechanical characteristics that can be experimentally determined. In fact, their high water retention accounts for this, as the extensive migration of water molecules through their porous matrix permits the stress to relax under a constant load, a phenomenon commonly referred to as poroelasticity (Chaudhuri, 2017; Caccavo et al., 2018). Nevertheless, in most investigations dealing with the role of time-dependent mechanical characteristics on cell response, hydrogels are treated as simple viscoelastic systems, while poroelastic properties are not incorporated. This simplification is tolerable provided that the solvent diffusion time is much larger than the time scales of viscoelastic relaxation and cellular processes under investigation (Caccavo et al., 2018).

### Theoretical Predictions of Tunable Viscoelasticity and Their Impact on Cellular Microenvironments

**FIGURE 6:**
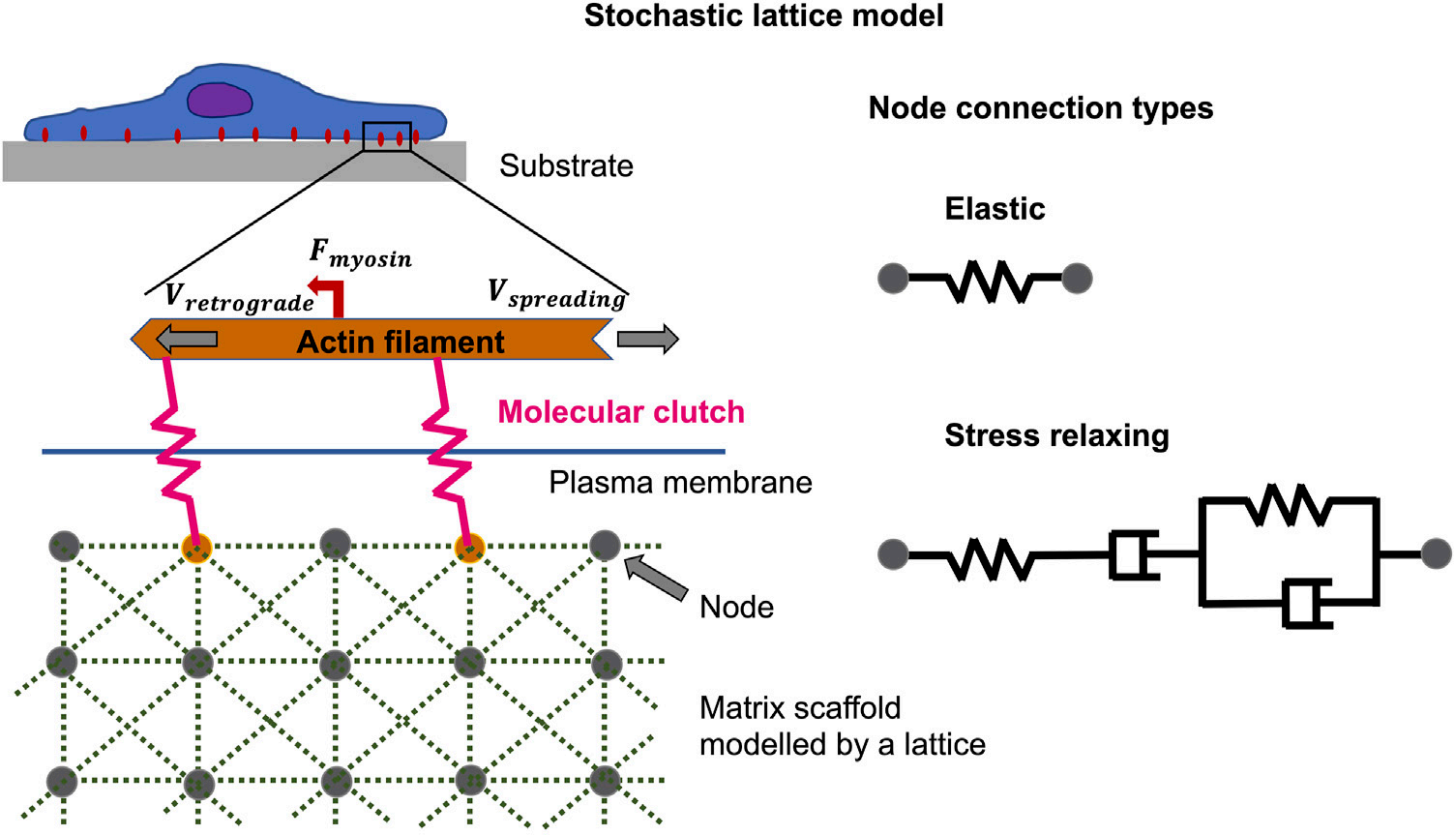
Non-equilibrium phenomena can be described by the stochastic lattice model, such as cell spreading on elastic or viscoelastic tumor microenvironments with low stiffness. The polymerization of actin at the cell front edge is tethered to the underlying substrate by molecular clutches that impede the retrograde flow of myosin motors acting on actin filaments. The elastic substrate is modelled by multiple nodes coupled by Hookean springs, whereas the viscoelastic substrate, which exhibits stress relaxation, is modelled by Burgers model elements.

The theoretical predictions involving analysis of the viscoelastic relaxation time scale are in outstanding accord with previous monitoring and furnish the groundwork for interpreting experiments in which extracellular matrices with tunable viscoelastic characteristics have been constructed by two distinct techniques (Chaudhuri et al., 2020). For example, to simulate focal adhesion binding and cell spreading characteristics, there are both analytical and Monte Carlo methods that can be employed. Instead of utilizing a stochastic lattice model (Figure 6) (Chaudhuri et al., 2015), a simple linear viscoelastic standard substrate has been combined with the motor-coupling model to illustrate how multiple viscoelastic material variables, including long-term stiffness, additional stiffness, and viscosity, adjust cell spreading. More specifically, viscosity at a low long-term and additional stiffness encourages cell spreading, and peak cell spreading is accomplished at an intermediate viscosity level. In contrary, the viscosity has a marginal influence on the spreading when the long-term stiffness is high. This unresponsiveness of cell spreading toward viscosity in this regime is due to the coupling enhancement phenomenon, which results in saturation of the limited couplings that can be built on stiff extracellular matrices. Such a strengthening mechanism (under high coupling load), nevertheless, may not be available in specific cell types such as neurons (Koch et al., 2012; Swaminathan and Waterman, 2016), eventually causing repressed cell spreading when the substrate turns very stiff.

Through the detection of the mechanism through which extracellular matrix viscoelasticity impacts cell spreading over a wide range of material properties, the analytical model by Chaudhuri seems to provide a valuable resource for the designing of biomaterials that maximize cellular adhesion and mechanosensing. Most notably, intermediate viscosity is determined to actually optimize cell spreading on soft substrates, whereas cell spreading on stiff substrates is not affected by viscosity. This knowledge could then be exploited to engineer dissipative biomaterials for optimized management of cellular performance (Gong et al., 2018). In parallel with stiffness, the viscosity of the extracellular matrix decisively effects the performance and functioning of cells. But the mechanism underlying such mechanosensitivity to viscoelasticity still is elusive. Thus, the evolution of motor coupling dynamics, such as focal adhesions, occurring between the cell and a viscoelastic substrate, has been systematically explored through analytical techniques and a direct Monte Carlo simulation. When the stiffness of the extracellular matrix is less, maximum cell spreading is obtained at an ideal viscosity level where the substrate relaxation time is intermediate between the coupling bond time scale and the characteristic bond lifetime. Specifically, viscosity acts to rigidify soft substrates on a time scale more rapid than the disengagement rate, which promotes cell adhesion to the extracellular matrix and increases cell spreading. Alternatively, for substrates that are rigid, this model predicts that viscosity has no effect on cell spreading because the bound couplings are at saturation due to the increased stiffness. The model has been validated and verified by experimental testing on three distinct material systems, and provides an interpretation of the varying observed implications of viscosity for each substrate. By grasping the mechanism through which substrate viscoelasticity governs how cells spread over a broad array of material properties, this analytical model offers a valuable guide for the construction of biomaterials for cancer research that maximize adhesion and mechanosensing of cells.

### Fine-Tuning of Tumor Extracellular Matrix’s Structure, Architecture and Mechanics, Including Viscoelasticity

The easiest way to fine-tune extracellular matrix models, such as collagen hydrogels, is to increase the concentration of collagen type I monomers. More intricate fine-tuning can be performed by adding specific cross-linkers that can be simply chemical cross-linkers, biomolecule-based cross-linkers or cell-derived cross-linkers. All of which varies the overall mechanical properties of tumor extracellular matrix scaffolds employed to explore cancer cell behaviors, such as adhesion and motility. For example, the nature of collective migration of cells has been seen to rely to varying degrees on the density (Gudipaty et al., 2017; Tlili et al., 2018) and motility (Bi et al., 2016; Hayer et al., 2016) of individual cells and intercellular adhesion (Benjamin et al., 2010; Vedula et al., 2012). For instance, in a confluent cell monolayer, an augmentation of cell motility can lead to a transition from a solid to liquid state (Hayer et al., 2016; Malinverno et al., 2017), whereas a breakdown of intercellular links can result in random uncorrelated cell movements (Benjamin et al., 2010; Vedula et al., 2012).

Apart from intrinsic characteristics of cells, extrinsic signals, including geometric confinement of the tumor microenvironment (Tanner et al., 2012; Vedula et al., 2012; Doxzen et al., 2013; Camley et al., 2014; Li and Sun, 2014; Segerer et al., 2015; Lin et al., 2017), chemical factors (Harris et al., 2012) and electric field (Cohen et al., 2014) can additionally impact dynamic properties of cells. Migrating cancer cells *in vivo* are frequently constrained geometrically by the tumor surrounding environment, such as extracellular matrix or other tumor stroma embedded cells. A typical case is the invasion of cancer cells within the porous peritumoral stroma (Friedl et al., 2012).

The main physical constraints faced by migrating cells *in vivo* are adhesion (friction), boundary, rigidity of migrating substrates, shear flow of extracellular liquids, topology and density of the ambient tissue or extracellular matrix scaffold (Charras and Sahai, 2014). To address these physical constraints of a 3D microenvironment, individual cells quickly change their viscoelasticity to recontour and “squeeze” or withstand deformation (Petrie and Yamada, 2012; Mueller et al., 2017). Nonetheless, to modify their viscoelastic characteristics and face their physical migratory microenvironment as a supracellular entity, cells within migratory aggregates must orchestrate the machinery that accomplishes such transformations.

Apart from the cell migratory aspect, the fine-tuning of the tumor extracellular matrix seems to be necessary to mimic the process of matrix alteration during cancer disease progression. In particular, the viscoelastic characteristics of hydrogels can be tailored in the course of time by agents that influence the forming or breaking down of cross-links. In addition, enzymatic crosslinkers use the reaction kinetics of the enzymes to change the viscoelastic characteristics of the gel with time (Mattei et al., 2020; Cacopardo and Ahluwalia, 2021). Slow kinetics chemical reactions have also been applied to produce gels with time-varying viscoelasticity (Guvendiren and Burdick, 2012; Rodell et al., 2015; Arkenberg et al., 2019; Carberry et al., 2020). Stem cell engagement switch that can be leveraged to stiffen hydrogels (Das et al., 2016). Consequently, the use of reactive materials is an interesting tactic to modify the viscoelasticity of gels as needed (Abdeen et al., 2016; Chen et al., 2021; Tran et al., 2021). For this purpose, these reactive materials have been shown to affect, for instance, the adhesion and spreading behavior of mesenchymal stem cells on magneto-responsive gels when the gel transitions from an elastic to a liquid-like characteristic (Abdeen et al., 2016). Most promising appear time evolving hydrogels. However, there is still too little research activity there. Therefore, further endeavors in this pursuit are required to develop mechano-mimetic approaches capable of replicating pathophysiological processes *in vitro*.

### Effect of Time Scales on Extracellular Matrix Scaffold’s Viscoelasticity

Special attention will be paid to the behavioral links between the various time scales concerned, such as mechanical, cellular, and observational, and to the principles of scaling that must be taken into account when developing viscoelastic materials and conducting tests for biomechanical or mechanobiological engineering purposes. The observational time scale is often not addressed and not included here. Time scale analysis identified that extracellular matrix viscoelasticity controls cell spreading, on the basis of the extent of the substrate relaxation time scale in comparison to the time scales of motor coupling binding and the focal adhesion lifespan. The coupling (clutch) binding time, τ_b_, is entirely due to the stochastic binding signature of the focal adhesion molecules, such as integrins, talin, paxillin and vinculin, with value of around 1 s (Chan and Odde, 2008; Bangasser and Odde, 2013). The interplay of myosin motor traction and substrate rigidity results in a focal adhesion lifetime timescale, τ_l_, ranging from 10^–1^ s to 10^3^ s. When no strengthening is acting, the focal adhesion lifetime is linear proportional to its lifetime time scale, τ_l_ (Bangasser and Odde, 2013). Average lifetimes vary from approximately 10 s–100 s, consistent with nascent focal adhesions within the lamellipodium, relying on stiffness (Choi et al., 2008). But once the enhancement is in effect, the focal adhesion lifetime could be in minutes or possibly beyond (a divergent condition), which is in agreement with a large number of experiments demonstrating robust focal adhesions for stiffer carriers (Walcott et al., 2011; Trichet et al., 2012; Cao et al., 2015). The substrate relaxation time scale, τ_s_, would encompass a broad spectrum from 10^–2^ s to 10^2^ s, which matches the viscoelastic character of diverse substrates. The behavioral association between three timescales, such as τ_l_, τ_b_, and τ_s_, distinctly accounts for how viscosity governs cell spreading. In particular, when τ_l_ > τ_b_, the spreading of cells is largest when the relaxation timescale τ_s_ lies between the binding timescale τ_b_ and the lifetime timescale τ_l_ (τ_b_ <τ_s_ <τ_l_) (Figure 5). However, when τ_l_ < τ_b_, the coupling reinforcement occurs and causes a saturation of the cell spreading area. Under this circumstance, the viscosity is not altering the spreading behavior of cells. The relaxation timescales, which are shorter than the coupling (binding) timescale (τ_s_ < τ_b_) exhibit a negligible impact on the spreading of cells.

Convenient way to identify the effective relaxation time scale of viscoelastic substrate in terms of its impact on cell spreading has been elaborated. Therefore, firstly, the relaxation time spectra need to be captured from stress relaxation data of viscoelastic materials. Secondly, the most prominent timescale, such as the highest peak for τ_s_ ≥ τ_b_, has been figured out of the relaxation spectrum as the so-called effective timescale. Computer simulations using multiple timescales revealed that the effective timescale perfectly mirrors the dynamical nature of the process of cell spreading on viscoelastic substrates. However, in instances where there are multiple prominent relaxation times past the binding time scale, the simulations indicate that the final outcome cell spreading is roughly a weighted average of the response for every time scale.

Earlier investigations proposed that the impact of substrate viscoelasticity on cell spreading is related to local substrate compaction and plastic flow (Chaudhuri et al., 2015; Bauer et al., 2017). Nevertheless, these experimental findings can be replicated by the newly proposed model (Chaudhuri et al., 2020), demonstrating that viscoelasticity by itself is fully adequate to account for the results obtained. Importantly, it is worth noting that this model is able to adequately clarify the viscoelastic control of cell spreading for three completely distinct types of hydrogels, including alginate, hyaluronic acid (HA) and polyacrylamide, with various manners of imparting viscoelasticity, such as supramolecular interactions, semi-interpenetrating entanglements of the scaffold and ionic-based cross-linking, distinct stiffnesses in the spectrum from 10^–1^ pN/nm to 10^1^ pN/nm, and various cell types, encompassing human MSCs, 3T3 fibroblasts and the U2OS osteosarcoma cell line. In addition, it is also possible to use this model for various cancer cell types. Moreover, experiments with substrates produced in various manners and displaying virtually no plasticity are also covered by the model predictions. These findings show that viscoelasticity by itself possesses a strong impact on cell spreading, plasticity and extracellular matrix restructuring that all seem to be equally critical. A more sophisticated model that encompasses the plastic theory may be addressed for future applications.

In summary, an analytical model incorporating the viscoelastic relaxation time range has successfully elucidated the implications of substrate viscoelasticity on cell spreading of various cell types and collagen matrices (Gautieri et al., 2013; Nam et al., 2016). This model can be employed as a theoretical framework for continued study of viscoelastic control of cell performance for multiple cancer cell types and tissues. It is also able to aid in forecasting cell spreading over the entire parameter range for viscoelastic substrates to be used in cancer research, thereby permitting the streamlined design of biomaterials. Finally, this result offers both physical glimpses and a practical approach to examine how cellular and material time scales intersect to adjust cancer cell performance.

### Separation of Mechanical Characteristics From Other Matrix Parameters

During the design of tumor microenvironments for mechanobiological investigations, it is essential to ensure that cells have appropriate topographical and biochemical cues in addition to mechanical constraints, and, especially in 3D, to ensure adequate room for cell growth. Yet, there has been limited emphasis on accounting for these interacting elements, potentially causing a false interpretation of the findings. Typical investigations involve benchmarking the performance of cells on 2D plastic substrates versus cells on 2D or 3D gels. Not merely the mechanical characteristics are distinct, but also features such as surface roughness, surface chemistry, and haptotactic information are to be distinguished (Alenghat and Ingber, 2002; Bettinger et al., 2009; Trappmann and Chen, 2013; Woods et al., 2017). For example, a tension platform for the analysis of the interplay between cancer cell phenotype and tumor extracellular matrix stiffness has been developed (Cassereau et al., 2015). Notably, the precise mechanical tailoring of the stiffness of collagen hydrogels while retaining a constant composition and porosity.

Numerous efforts are devoted to the decoupling of stiffness and ligand density (A. Engler et al., 2004; Grevesse et al., 2013; S. J. Han et al., 2012), whereas fewer efforts are directed to stiffness and topography (Ahmed et al., 2021) or mineral grade (Mattei et al., 2020). The problem of decoupling interacting effects in mechotransduction is amplified in 3D gels because raising the polymer concentration and crosslinking not only modifies the mechanical response of the gels, but also impacts oxygen and nutrient diffusion as well as cell volume (García, 2014; Xue et al., 2021). Consequently, alterations in cell responsiveness can be affected or sometimes even obscured by issues other than the mechanical characteristics of the cell surrounding environment (Vandrangi et al., 2014). Currently, a particular difficulty *in vitro* research is to isolate or decouple mechanical characteristics from other quantities of parameters to completely comprehend and control cell performance by tailoring environmental factors that can be engineered and monitored.

## Final Remarks and Future Perspectives

How the tumor extracellular matrix shields, protects and nourish the primary tumor is important to fully understand the progression of cancer and the failure of cancer therapies in specific cancer types or distinct patients. A reason for this may be the diverse extracellular matrices of tumors. Multiple time, it has been shown that the extracellular matrix in tumors is pronouncedly altered in composition and structure compared to normal healthy tissue. As for their physical characteristics, the extracellular matrix of the tumor is richer, denser and stiffer. These modified characteristics may adversely impact the responsiveness to therapy in several respects. Apparently, exaggerated clustering of dense and rigid extracellular matrix, which histologically frequently envelops clusters of cancer cells, is able to function as a border barrier, insulating the cells from therapeutic compounds.

This specific behavior is directly related to a decreased overall perfusion, as this barrier also impedes the diffusion of oxygen, nutrients, and metabolites. Therefore, the tumor microenvironment limits the trafficking of oxygen and other nutrients that subsequently leads to immunosuppression in the primary tumor and later in the whole organism. A major reason for this is that the poorly functioning blood vessel of tumors are leaky and compressed, and some endothelial cells are replaced by cancer cells, which take over the role of endothelial cells. All of this must be reversed by potentially altering the mechanical factors in the tumor extracellular matrix to improve oxygenation of primary tumors. Therefore, immunotherapies should be combined with therapies that normalize the tumor microenvironment to synergistically augment oxygen transport and treatment outcomes. Elevated hypoxia and metabolic stress cause activation of anti-apoptotic and drug-resistant signaling cascades. Consequently, cell-extracellular matrix contacts and augmented tissue stiffness may participate in direct support of tumor chemoresistance through integrin and FAK signal transduction pathways. For the progress in the development of viscoelastic tumor microenvironment mimicking hydrogels, the time-evolving viscoelasticity needs to be addressed. Time-evolving viscoelasticity, which means how viscoelasticity alters during various stages of cancer, is a key aspect in mimicking the tumor microenvironment (Mattei et al., 2020).

Viscoelasticity of the migratory microenvironment is crucial to induce cell migration, engineer a material that enables the efficient migration of cells, and/or to regulate migration through the mechano-sensing-based process of durotaxis. Consistent with this, new advances have been explored that highlight possible mechanisms facilitating the transfer of mechanical cues from the tumor extracellular matrix environment into cells, their impact on the expression of traditional transcriptional controllers of epithelial-to-mesenchymal transition (EMT) of individual cancer cells, and their implications for altering the viscoelastic phenotype of migrating cells and their local tumor microenvironment. In this regard, the time-evolving viscoelastic alterations of hydrogels seem to be most promising and need future research effort.

Tissue interferences occur, including that the EMT, and offer the integration of morphogenesis as a mechano-molecular feedback circuit that coordinates the timing of cellular redistributions and gene expression patterns that are needed for cancer progression. Moreover, it has to be accounted for that molecular signal transduction causes cellular remodeling events, which alter the tissue including its viscoelastic characteristics, and this new viscoelasticity of the tissue environment can then act on a long-range timescale to alter the cellular, molecular, and viscoelastic characteristics of a neighbor tissue. This behavior is similar to a mechano-molecular feedback circuit that governs the process of morphogenesis. Since tissue interferences during morphogenesis are seen at a chemical scale through the secretion of molecules, it would be promising to investigate the interaction of viscoelasticity and secreted molecules in the regulation of collective movement of cancer cells, as an element of this integrative approach in cancer research. The involvement of these types of mechano-molecular feedback interferences seem to be highly crucial in the advancement for the forming and engineering of organ-analogous structures, such as organoids, for investigating the malignant progression of cancer and its mutual interplay with the tumor extracellular matrix scaffold. Thus, it is even more important to combine the analysis of local tumor microenvironment with molecular elements, viscoelastic variables of cells and gene expression patterns (Poh et al., 2014; Wrighton and Kiessling, 2015; Mierke, 2019).

Moreover, if it is known how these feedback loops act on the progression of cancer, the knowledge could be used to perform effective cancer therapies. Employing *in vivo* rat cancer metastasis models, the mechanical stress generated through gastric cancers toward their microenvironment has been seen to cause severe molecular impacts that are associated with a poor prognosis (Fernández-Sánchez et al., 2015). Identification of viscoelastic characteristics of wounds and inflammatory tissues together with the knowledge how these alterations impact the collective migration and cellular fate, may be beneficial in developing new therapies targeting these kinds of processes during the progression of cancer. Consequently, viscoelasticity represents a general characteristic feature for the vast majority of biological substances and the majority of cells and tissues that experience one or even another mechanical force. Lastly, the requirement of multidisciplinary studies combining biophysical and biochemical variables seem to be critical to obtain a knowledge of growing intricate living biological systems under diseased conditions, such as cancer.

Finally, it is important not only to address the stiffness of the tumor extracellular matrix network, but also to explore the viscoelastic characteristics of these networks that then act on the other mechanical characteristics, such as the aforementioned stiffness.
